# Driveline Infections in Durable LVAD Support: Risk Factors, Microbiology, and Resistance Patterns from a Large Cohort

**DOI:** 10.3390/diagnostics16091303

**Published:** 2026-04-27

**Authors:** Umit Kahraman, Oguzhan Acet, Barkin Dost Bulut, Aysen Yaprak Kapkın, Osman Nuri Tuncer, Meltem Tasbakan, Sanem Nalbantgil, Emrah Oguz, Cagatay Engin, Mustafa Ozbaran, Tahir Yagdi

**Affiliations:** 1 Department of Cardiovascular Surgery, Faculty of Medicine, Ege University, 35100 Izmir, Turkey; umitkahraman81@gmail.com (U.K.); barkin.dost.bulut@ege.edu.tr (B.D.B.); yaprak.engin2009@gmail.com (A.Y.K.); osnutuncer@gmail.com (O.N.T.); emrah.oguz@ege.edu.tr (E.O.); cagatay.engin@ege.edu.tr (C.E.); mustafa.ozbaran@ege.edu.tr (M.O.); 2 Department of Infectious Diseases, Faculty of Medicine, Ege University, 35100 Izmir, Turkey; oguzhan.acet@ege.edu.tr (O.A.); tasbakan@yahoo.com (M.T.); 3 Department of Cardiology, Faculty of Medicine, Ege University, 35100 Izmir, Turkey; sanem.nalbantgil@ege.edu.tr

**Keywords:** driveline infection, left ventricular assist device, antimicrobial resistance

## Abstract

**Background**: Driveline infection (DLI) is the most common device-specific infection in patients supported with ventricular assist devices (VADs) and remains a major cause of morbidity during long-term mechanical circulatory support. This study aimed to evaluate the incidence, risk factors, microbiological characteristics, and antimicrobial resistance patterns of DLIs in patients undergoing durable left ventricular assist device (LVAD) implantation. **Methods**: This retrospective cohort study included 772 consecutive patients who underwent durable LVAD implantation at a single tertiary center between January 2012 and December 2024. Patients were categorized according to the development of DLI: the DLI group (*n* = 158) and the non-DLI group (*n* = 614). Demographic, clinical, laboratory, perioperative, and postoperative variables were analyzed. Multivariate logistic regression analysis was performed to identify independent predictors of DLI. Microbiological isolates and antimicrobial resistance patterns were also evaluated. **Results**: Driveline infection developed in 20.5% of patients during follow-up. Patients with DLI had a significantly higher body mass index (26.4 vs. 24.8 kg/m^2^, *p* = 0.002) and a higher prevalence of diabetes mellitus (28.2% vs. 12.1%, *p* < 0.001). In multivariate analysis, diabetes mellitus (OR 3.29, *p* = 0.013) and longer LVAD support duration (*p* = 0.003) were independently associated with DLI. Device type showed differences in crude infection rates but was not an independent predictor. The most frequently isolated pathogens were *Staphylococcus aureus* (36%) and *Pseudomonas aeruginosa* (19%). The most common antimicrobial resistance patterns included fluoroquinolone resistance (23%), methicillin-resistant *Staphylococcus aureus* (10%), and resistance to piperacillin/tazobactam and carbapenems. **Conclusions**: In this large single-center cohort, diabetes mellitus and prolonged device support duration were the main independent predictors of driveline infection. *Staphylococcus aureus* and *Pseudomonas aeruginosa* were the predominant pathogens, with notable antimicrobial resistance patterns. These findings highlight the importance of metabolic optimization, meticulous driveline exit-site care, and structured long-term surveillance strategies for reducing infection risk in LVAD recipients.

## 1. Introduction

Ventricular assist devices (VADs) are an established therapy for patients with end-stage heart failure, leading to improved functional capacity, symptom relief, and quality of life [1]. VADs are commonly implanted as a bridge to transplant (BTT); however, an increasing proportion of patients who are ineligible for transplantation receive VADs as destination therapy (DT). Despite advances in device technology and perioperative management, VAD implantation is still associated with significant complications, including pump thrombosis, bleeding, cerebrovascular accidents (CVA), and infection [2,3].

Infection remains one of the most frequent and serious complications, affecting up to 60% of VAD recipients, and is associated with impaired quality of life, recurrent hospitalizations, and worse prognosis [4,5]. Among these, percutaneous driveline infections (DLIs) represent the most common form of VAD-specific infection and may progress to deeper infections involving the pump pocket, cannula, or device itself [6]. DLIs are prone to relapse, often necessitating repeated hospital admissions and prolonged antimicrobial therapy, thereby substantially increasing long-term healthcare costs [7,8].

Infections in VAD recipients are classified as VAD-specific, VAD-related, or non-VAD-related. VAD-specific infections directly involve device components such as the pump, cannula, pocket, or driveline, and may result from intraoperative contamination, colonization of the driveline exit site, or hematogenous seeding during bloodstream infections. Driveline infections are further categorized as superficial or deep, depending on whether the infection is limited to the skin and subcutaneous tissue or extends beyond the fascial and muscular layers. Trauma to the driveline exit site is a significant predisposing factor for infection development. Additionally, microbial adherence and biofilm formation on driveline surfaces play a central role in the pathogenesis of these infections. Microorganisms can adhere to both smooth and velour segments of the driveline, migrate along the subcutaneous tunnel, and establish biofilms that protect them from host immune responses and limit antibiotic penetration. These mechanisms contribute to persistent or recurrent infections that are often difficult to eradicate without combined medical and surgical management [9,10,11].

Staphylococcus species are the most frequently isolated pathogens in VAD-specific infections, followed by *Pseudomonas aeruginosa*, with other Gram-negative bacteria and *Candida* species also implicated [12].

In this study, we aimed to investigate driveline infections as a subset of VAD-specific infections in our patient population. The diagnosis of DLI was based on microbiological findings from swab and tissue cultures, clinical presentation, infection-related laboratory parameters, and infectious disease consultations. Owing to limited data distinguishing superficial from deep infections, all patients treated medically or undergoing surgical driveline revision were classified as having DLI.

This study seeks to identify risk factors associated with driveline infections, characterize causative microorganisms, evaluate antibiotic treatment strategies, and analyze antimicrobial resistance patterns in VAD recipients.

## 2. Materials and Methods

This retrospective cohort study included consecutive patients who underwent durable left ventricular assist device (LVAD) implantation at the Ege University Cardiovascular Surgery Clinic between January 2012 and December 2024. A total of 772 patients were analyzed. Patients were categorized into two groups according to the development of driveline infection (DLI): those who developed DLI (*n* = 158) and those who did not (*n* = 614).

This study included all consecutive adult patients who underwent durable LVAD implantation during the study period. Patients who underwent biventricular assist device implantation or temporary circulatory support were excluded. In addition, patients with incomplete medical records or insufficient follow-up data to determine driveline infection status were also excluded from the analysis.

The study protocol was approved by the Ege University Ethics Committee (approval number: 23-5.1T/3), and the study was conducted in accordance with the principles of the Declaration of Helsinki.

Demographic, clinical, laboratory, perioperative, and postoperative data were retrospectively extracted from institutional electronic medical records.

Baseline variables included age, sex, body mass index (BMI), hypertension, diabetes mellitus, hyperlipidemia, prior cardiac surgery, and comorbidities such as gastrointestinal bleeding, chronic obstructive pulmonary disease, and prior cerebrovascular events.

Preoperative laboratory parameters included serum creatinine, albumin, hemoglobin, international normalized ratio (INR), lactate, and leukocyte count. Heart failure severity was assessed using the Interagency Registry for Mechanically Assisted Circulatory Support (INTERMACS) classification. Additional preoperative variables included transpulmonary gradient, N-terminal pro-brain natriuretic peptide (NT-proBNP) levels, preoperative inotropic support, intra-aortic balloon pump (IABP) use, and extracorporeal membrane oxygenation (ECMO) support.

Surgical characteristics included the surgical approach (median sternotomy, thoracotomy, or minimally invasive techniques), the type of implanted durable LVAD, cardiopulmonary bypass (CPB) duration, aortic cross-clamp time, and concomitant surgical procedures (tricuspid valve repair, aortic valve replacement, or aortic replacement). Re-exploration due to postoperative bleeding was recorded.

Postoperative variables included the development of right heart failure, the use of inhaled nitric oxide, prolonged inotropic support (>14 days), requirement for temporary right ventricular assist device (RVAD) support (Levitronix^®^ (Levitronix Technologies Inc., Zurich, Switzerland) or ECMO), and long-term RVAD support. Postoperative complications included acute renal failure, need for hemodialysis, sepsis, and length of intensive care unit (ICU) stay.

Long-term outcomes included gastrointestinal bleeding, ischemic and hemorrhagic stroke, pump thrombosis, the duration of LVAD support, mortality, and heart transplantation.

The diagnosis of driveline infection was established based on clinical findings (erythema, purulent discharge, local pain, or abscess formation at the exit site), microbiological culture results obtained from the driveline region, infection-related laboratory parameters, and multidisciplinary evaluation including consultation with the infectious diseases department.

Infection definitions were based on the 2024 consensus statement of the International Society for Heart and Lung Transplantation (ISHLT) for mechanical circulatory support-associated infections. Infections were classified as device-specific, device-related, and non-device-related infections. Device-specific infections included driveline, pump, cannula, and pocket infections. Driveline infection (DLI) was defined as infection involving the percutaneous driveline exit site or subcutaneous tunnel, diagnosed based on clinical signs and symptoms such as erythema, purulent discharge, local tenderness, or abscess formation, and supported by microbiological evidence and/or imaging findings when clinically indicated. Driveline infections were further categorized as superficial, limited to the skin and subcutaneous tissue, or deep, extending beyond the fascial or muscular layers. Device-related infections included bloodstream infections and endocarditis associated with the device, whereas non-device-related infections were defined as infections not directly related to the mechanical circulatory support system.

Microbiological samples included driveline exit-site swabs, tissue or biopsy specimens, and abscess material when present. When clinically indicated, additional cultures (blood, urine, sputum, or deep tracheal aspirates) were obtained to exclude systemic infection.

Due to incomplete documentation distinguishing superficial from deep driveline infections, all patients who received targeted medical treatment for DLI or underwent surgical driveline revision were classified as having driveline infection. Among the 158 patients with DLI, 10 underwent surgical driveline relocation or revision. All patients received standardized antimicrobial therapy.

Exit-site care in our institution consisted of daily sterile dressing changes using chlorhexidine-based antiseptic solutions.

At our center, empirical therapy for suspected driveline infections is guided by the most recent cumulative antibiogram data, individual patient characteristics, prior microbiological results, and clinical severity at presentation. Particular consideration is given to risk factors for multidrug-resistant (MDR) pathogens, including prior broad-spectrum antibiotic exposure, prolonged or repeated hospitalization, intensive care unit (ICU) stay, previous colonization or infection with resistant organisms, and recent device-related interventions.

In clinically stable patients without MDR risk, empirical therapy consists of a beta-lactam active against methicillin-susceptible *Staphylococcus aureus* (MSSA) and *Enterobacterales* spp. In patients presenting with severe infection, prior broad-spectrum antibiotic exposure, prolonged hospitalization, or known colonization with resistant organisms, broader anti-pseudomonal coverage is initiated. Empirical coverage against methicillin-resistant *Staphylococcus aureus* (MRSA) and *methicillin-resistant coagulase-negative staphylococci* (MR-CoNS) is added when clinically or epidemiologically indicated.

Antimicrobial therapy is reassessed within 48–72 h and de-escalated according to microbiological results, with surgical source control performed when necessary.

Isolated microorganisms were identified using standard institutional microbiological techniques. Antimicrobial susceptibility testing was performed according to institutional laboratory protocols, and resistance patterns of isolated pathogens were recorded and analyzed.

Episode-level infection data were not consistently available for all patients, limiting formal recurrence and time-to-reinfection analyses.

### 2.1. Statistical Methods

Statistical analyses were performed using the IBM Statistical Package for the Social Sciences (SPSS) for macOS, version 30.0 (IBM Corp., Armonk, NY, USA). Categorical variables were expressed as frequencies and percentages, while continuous variables were presented as mean ± standard deviation or median (minimum–maximum), as appropriate. The normality of continuous variables was assessed using the Kolmogorov–Smirnov test.

For intergroup comparisons, the Mann–Whitney U test was used for continuous variables, and the chi-square test or Fisher’s exact test was applied for categorical variables. A *p*-value < 0.05 was considered statistically significant.

### 2.2. Logistic Regression Analysis

Multivariate logistic regression analysis was performed to identify independent factors associated with driveline infection. Variables considered clinically relevant, as well as those associated with DLI in univariate analyses (*p* < 0.10), were included as candidate variables. Age and sex were entered into the model as principal covariates. Variables with no events in the DLI group were excluded to ensure model stability.

The final model included age, sex, body mass index, diabetes mellitus, device type, reoperation due to bleeding, postoperative nitric oxide use, length of stay in the intensive care unit, and total duration of device support. All selected variables were entered simultaneously into the model using the enter method to maintain clinical interpretability. Adjusted odds ratios (ORs) with corresponding 95% confidence intervals (CIs) were reported, and statistical significance was defined as *p* < 0.05.

## 3. Results

A total of 641 patients (83%) were male and 131 (17%) were female. Among patients who developed driveline infection, 126 (79.7%) were male and 32 (20.3%) were female. The mean age was 53 years in both groups. Mean body mass index was significantly higher in patients with driveline infection compared with those without infection (26.4 vs. 24.8, *p* = 0.002).

Dilated cardiomyopathy was the most common underlying diagnosis in both groups, observed in 318 patients (52.9%) without driveline infection and 80 patients (51%) with driveline infection. Diabetes mellitus was significantly more prevalent among patients with driveline infection (28.2% vs. 12.1%, *p* < 0.001). Hyperlipidemia was more frequently observed in patients with driveline infection (12.8% vs. 6.2%, *p* = 0.009).

Other evaluated baseline characteristics, including peripheral arterial disease, chronic obstructive pulmonary disease, gastrointestinal bleeding, cerebrovascular accident, preoperative hemodialysis, and preoperative laboratory parameters (pro-BNP, creatinine, bilirubin, INR, albumin, hemoglobin, and leukocyte count), did not differ significantly between groups. Baseline demographic and clinical characteristics are summarized in Table 1.

No significant differences were observed between groups regarding prior cardiac surgery, preoperative lactate levels, preoperative inotropic support, INTERMACS classification, preoperative intra-aortic balloon pump use, or preoperative extracorporeal membrane oxygenation support (Table 2).

A significant difference in driveline infection rates was observed according to device type. Driveline infection occurred in approximately 18% of patients with the HeartWare (Medtronic, Inc., Minneapolis, MN, USA) (HVAD), 22% of patients with the HeartMate II (Abbott Inc., Abbott Park, IL, USA), and 34% of patients with the HeartMate 3 (Abbott Inc., Abbott Park, IL, USA) device (Table 3).

Comparison of surgical characteristics, including surgical approach (sternotomy, thoracotomy, or minimally invasive techniques), cardiopulmonary bypass duration, aortic cross-clamp time, and concomitant surgical procedures (tricuspid valve repair, aortic valve replacement, or aortic replacement), revealed no statistically significant differences between groups (Table 4).

Reoperation due to postoperative bleeding occurred in 9.2% of patients without driveline infection and 3.8% of patients with driveline infection. Postoperative nitric oxide use was observed in 20.5% of patients without driveline infection and 10.8% of those with driveline infection. Temporary postoperative RVAD support (Levitronix^®^ (Levitronix Technologies Inc., Zurich, Switzerland) or ECMO) and postoperative hemodialysis were observed only in patients without driveline infection.

The median length of stay in the intensive care unit was significantly longer in patients with driveline infection (5 [2–60] days) compared with those without infection (5 [1–60] days; *p* = 0.038). Total duration of LVAD support was also significantly longer in patients with driveline infection (1995.5 vs. 870.5 days, *p* < 0.001).

No statistically significant differences were observed between groups regarding postoperative right heart failure, prolonged inotropic support (>14 days), long-term RVAD support, postoperative complications (acute renal failure, sepsis), or long-term complications, including gastrointestinal bleeding, ischemic stroke, hemorrhagic stroke, and pump thrombosis (Table 5).

Multivariate analysis demonstrated that diabetes mellitus was independently associated with driveline infection, conferring approximately a threefold increased risk (*p* = 0.013) and longer device support duration was significantly associated with increased infection risk (*p* = 0.003). Higher body mass index also showed a trend toward increased risk (*p* = 0.066). Age, sex, device type, reoperation due to bleeding, nitric oxide use, and intensive care unit length of stay were not independently associated with driveline infection (Table 6).

Among patients with driveline infection, 79.7% (*n* = 126) were male and 20.3% (*n* = 32) were female. A total of 1624 microbiological samples were obtained from 158 patients. Swab cultures constituted the most common sample type (63.7%, *n* = 1035) (Table 7).

The most frequently isolated pathogens were *Staphylococcus aureus* (36%), followed by *Pseudomonas aeruginosa* (19%), *Escherichia coli* (6%), *Klebsiella pneumoniae* (6%), and *Corynebacterium striatum* (3%). Ciprofloxacin (fluoroquinolone) resistance was observed in 23% of isolates, methicillin-resistant *Staphylococcus aureus* (MRSA) in 10%, piperacillin/tazobactam (penicillins, including beta-lactamase inhibitors) resistance in 10%, penicillin resistance in 9%, and imipenem (Carbapenem) resistance in 8% (Table 8).

In addition to patient-level analysis, infection burden was further evaluated in terms of recurrent infection-related hospitalizations and episode frequency. A total of 89 patients (11.5% of the entire cohort) experienced recurrent driveline infection-related hospitalizations. Overall, 234 infection episodes were identified during the follow-up period, corresponding to 0.076 episodes per patient-year based on 3070 patient-years of observation. Therefore, a formal time-to-reinfection or recurrent-event analysis was not performed due to insufficiently reliable episode-level data.

### Surgical Outcomes of Patients with Deep Driveline Infection

A total of 10 patients with deep driveline infections requiring surgical intervention were analyzed. The majority of patients were male (8/10), with a median age of 43 years. Diabetes mellitus was present in two patients. The time to infection varied widely, ranging from 10 days to 29 months. All patients had received prior antibiotic therapy; however, conservative management failed to achieve infection control. Clinically, all patients presented with drainage and tenderness, while erythema was observed in most patients (8 patients) and soft tissue infiltration in some (5 patients). The infection was predominantly localized (9/10), with only one patient exhibiting systemic signs.

All patients underwent surgical debridement. Deep tissue involvement (8 patients) and driveline exposure (9 patients) were identified in the majority of cases. Additionally, flap reconstruction was performed in six patients, and driveline repositioning in five patients. Negative Pressure Wound Therapy (NPWT) was used in a limited number of patients. Microbiologically, the most common pathogen was Methicillin-Susceptible *Staphylococcus aureus* (MSSA) (7 patients), followed by *Pseudomonas aeruginosa* (2 patients) and *Serratia marcescens* (1 patient).

In the postoperative period, local surgical complications (bleeding, hematoma) occurred in only three patients. Eight patients required hospital readmission, and six patients underwent reoperation.

## 4. Discussion

Driveline infection (DLI) remains the most common device-specific infection in patients supported with ventricular assist devices (VADs). Occurring at the percutaneous exit site of the driveline connecting the implanted pump to the external controller, DLI is associated with substantial morbidity and mortality. Management remains challenging due to difficulties in achieving adequate source control, particularly in the presence of biofilm formation and antimicrobial resistance, which often lead to recurrent or chronic infection requiring prolonged suppressive therapy, surgical revision, pump exchange, or transplantation. Although early postoperative vulnerability is recognized, cumulative exposure over time significantly increases infection risk [11,13].

Previously described risk factors include younger age, obesity, diabetes mellitus, chronic kidney disease, depression, longer duration of VAD support, lower cardiac index, advanced heart failure severity, and mechanical trauma at the driveline exit site [11].

In our study, body mass index (BMI) was significantly higher among patients who developed DLI. Although BMI did not remain an independent predictor in multivariate analysis, it demonstrated a trend toward increased risk. Obesity may impair wound healing, increase mechanical stress at the exit site, and facilitate bacterial colonization, findings consistent with prior reports [14].

Diabetes mellitus was significantly more prevalent in the DLI group. Multivariate analysis confirmed diabetes mellitus as an independent predictor of driveline infection. Given the well-established effects of chronic hyperglycemia on immune dysfunction, impaired angiogenesis, reduced tissue perfusion, and delayed wound healing, diabetes represents a clinically relevant contributor to DLI susceptibility [15,16]. These findings underscore the importance of strict glycemic control in VAD recipients.

Hyperlipidemia was more frequent among patients with DLI (12.8% vs. 6.2%, *p* = 0.009). Although its effect was attenuated after adjustment for confounders, impaired tissue perfusion, chronic inflammation, and delayed healing associated with dyslipidemia may contribute to exit-site vulnerability.

Thromboembolic events remain major complications in VAD recipients. Data from the Interagency Registry for Mechanically Assisted Circulatory Support (INTERMACS) have shown a higher prevalence of cerebrovascular accidents in patients with active infection compared with those without infection [17]. However, we did not observe a significant association between DLI and cerebrovascular events. Differences in infection timing, definitions, and study design may explain this discrepancy, suggesting that localized driveline infection alone may not independently increase cerebrovascular risk.

Gastrointestinal bleeding is another well-recognized complication in LVAD recipients, with cumulative incidences of 18%, 23%, and 27% at 1, 2, and 3 years post-implantation, respectively, according to INTERMACS reports [18]. Although infection may theoretically influence coagulation balance and INR variability in patients receiving warfarin and acetylsalicylic acid, current evidence does not support a direct causal relationship between DLI and gastrointestinal bleeding. Consistent with the literature, we found no significant association.

Preoperative clinical and laboratory parameters—including peripheral arterial disease, chronic obstructive pulmonary disease, pro-BNP, bilirubin, INR, albumin, hemoglobin, leukocyte count, creatinine, and dialysis requirement—were not independently associated with DLI [19]. Similarly, preoperative INTERMACS classification did not predict infection risk, supporting the concept that DLI is multifactorial rather than determined by isolated baseline variables [13].

Redo cardiac surgery was not associated with DLI in our cohort. Although repeated procedures increase device-related infection risk in pacemaker and ICD populations [20], this relationship has not been clearly demonstrated in VAD recipients. Likewise, preoperative lactate levels, inotropic support, intra-aortic balloon pump (IABP) use, and extracorporeal membrane oxygenation (ECMO) were not significant predictors, indicating that markers of hemodynamic instability do not specifically predispose patients to DLI [21,22,23].

Device type appeared to influence infection rates. DLI incidence was approximately 18% with HeartWare (Medtronic, Inc., Minneapolis, MN, USA), 22% with HeartMate 2 (Abbott Inc., Abbott Park, IL, USA), and 34% with HeartMate 3 (Abbott Inc., Abbott Park, IL, USA) in our cohort. Meta-analytic data have reported weighted mean DLI rates ranging from 7.2% for HeartMate 2 (Abbott Inc., Abbott Park, IL, USA) to 11.9% for HeartMate 3 (Abbott Inc., Abbott Park, IL, USA) [24], and single-center studies have similarly suggested device-specific differences [25,26]. The higher incidence observed in HeartMate 3 (Abbott Inc., Abbott Park, IL, USA) recipients may relate to driveline design characteristics, including a heavier and modular structure that may complicate stabilization [27]. Differences in implantation era and support duration may also contribute and should be considered when interpreting these findings.

Intraoperative variables—including surgical approach, cardiopulmonary bypass time, cross-clamp time, and concomitant procedures—were not associated with DLI, suggesting that infection risk is primarily influenced by patient-related and local exit-site factors rather than operative technique.

Postoperative factors demonstrated mixed findings. Reoperation for bleeding, nitric oxide therapy, temporary RVAD support (including Levitronix^®^ (Levitronix Technologies Inc., Zurich, Switzerland) or ECMO), and postoperative hemodialysis were not associated with increased DLI risk [28,29]. Although patients requiring dialysis are often critically ill, neither prior literature nor our data support a predisposing effect.

Prolonged ICU stay was associated with DLI. Although median ICU duration was similar between groups, patients who developed infection demonstrated a broader distribution with a higher proportion of extended stays (*p* = 0.038), supporting prior observations linking prolonged hospitalization to infection risk [30].

Duration of VAD support emerged as one of the strongest predictors of DLI. Patients with infection had significantly longer support times, and multivariate analysis confirmed total support duration as an independent predictor (*p* = 0.003). Previous studies have demonstrated increased infection incidence beyond 90–180 days, with a median time to infection of approximately 6.5 months [30,31]. These findings reinforce the cumulative temporal risk associated with prolonged device support.

Right heart failure, prolonged inotropic therapy (≥14 days), and postoperative sepsis were not independently associated with DLI. Although DLI may coexist with sepsis in a substantial proportion of cases [29], we did not demonstrate a statistically significant relationship, possibly reflecting differences in timing and event classification.

VAD-related infections have also been linked to adverse neurological outcomes. In a cohort of 402 patients, VAD infection occurred in 39% of cases, with driveline infections accounting for 17% [32]. Infection-related stroke was observed in 13% of infected patients; ischemic stroke was associated with wound infection, and hemorrhagic stroke was associated with bloodstream infection, while no association was found with driveline or pump pocket infection. Other studies have shown that VAD infections, particularly bloodstream infections, are associated with pump thrombosis, bleeding complications, prolonged hospitalization, reduced transplantation rates, and increased mortality [33]. Although small case series have suggested a possible link between active driveline infection and intracranial hemorrhage [34], our study did not identify an association between driveline infection and long-term complications such as gastrointestinal bleeding, ischemic stroke, hemorrhagic stroke, or pump thrombosis.

Microbiologically, our findings align with previous reports [35,36,37,38,39]. *Staphylococcus aureus* (36%) and *Pseudomonas aeruginosa* (19%) were the most frequently isolated pathogens. Resistance patterns included fluoroquinolone resistance (23%), methicillin-resistant *Staphylococcus aureus* (10%), and resistance to piperacillin/tazobactam and imipenem. These data underscore the ongoing challenge of antimicrobial resistance and biofilm-associated infection in VAD recipients.

## 5. Conclusions

In our cohort, prolonged device support duration and diabetes mellitus independently emerged as the most consistent predictors of driveline infection. *Staphylococcus aureus* and *Pseudomonas aeruginosa* were the predominant pathogens, and fluoroquinolone resistance represented the most frequent antimicrobial resistance pattern. Within the limits of this retrospective analysis, isolated driveline infection was not associated with major long-term adverse events. These findings emphasize the importance of metabolic optimization, meticulous exit-site management, and structured long-term surveillance strategies in patients receiving durable VAD support.

## 6. Limitations

This study has several limitations that should be acknowledged. First, its retrospective and single-center design inherently limits causal inference and introduces potential selection bias and missing data. In addition, the long study period (2012–2024) and heterogeneity in device types and management strategies used may have introduced variability in outcomes and clinical practice patterns.

Second, detailed information regarding driveline exit-site care, including hygiene adherence, dressing change frequency, stabilization techniques, and local trauma, was not systematically recorded and therefore could not be included in the analysis. Similarly, data on glycemic control and metabolic status (e.g., HbA1c levels and perioperative glucose variability) were incomplete, limiting evaluation of the role of diabetes-related metabolic control in infection risk.

Third, due to incomplete retrospective documentation, superficial and deep driveline infections could not be consistently distinguished. Therefore, classification relied on clinical, microbiological, laboratory, and infectious disease consultation data. While this approach may have led to the potential overestimation of infection incidence, it reflects real-world clinical decision-making.

Fourth, in a subset of patients, the exact timing of initial or recurrent driveline infections could not be reliably determined. Additionally, some patients initially treated at our center were later followed at external institutions and subsequently re-admitted. As a result, time-to-event analyses such as Kaplan–Meier and Cox regression could not be performed, limiting assessment of temporal trends and era effects.

Fifth, some infection episodes were inconsistently documented, which limited our ability to clearly distinguish whether infections represented persistent, relapsing, or separate new episodes. Consequently, formal recurrent-event analyses could not be conducted. Moreover, detailed longitudinal data on antibiotic duration, treatment sequencing, and relapse outcomes were not consistently available, limiting evaluation of treatment-specific effects.

Despite these limitations, this study represents one of the largest single-center experiences evaluating driveline infections over a long-term follow-up period and provides valuable real-world data on risk factors, microbiology, and outcomes in VAD patients.

## Figures and Tables

**Table 1:** Patients’ diagnosis, comorbidities, preoperative demographics, and laboratory data.

	Total	No Driveline Infection (*n* = 614)	with Driveline Infection (*n* = 158)	*p*-Value
	*n* (%) or Median (Min–Max)	*n* (%) or Median (Min–Max)	*n* (%) or Median (Min–Max)	
Age	53 (7–74)	53 (7–74)	53 (7–71)	0.694
Gender				0.217
Male	641 (83)	515 (83.9)	126 (79.7)	
Female	131 (17)	99 (16.1)	32 (20.3)	
Body mass index (BMI)	25.3 (6.4–37.9)	24.8 (6.4–37.9)	26.4 (6.4–37.9)	0.002
Ischemic cardiomyopathy (ICM)	357 (47.1)	280 (46.6)	77 (49)	0.583
Dilated cardiomyopathy (DCM)	398 (52.5)	318 (52.9)	80 (51)	0.662
Arrhythmogenic right ventricular dysplasia (ARVD)	4 (0.5)	3 (0.5)	1 (0.6)	1.000
Restrictive cardiomyopathy (RCM)	2 (0.3)	2 (0.3)	0 (0)	1.000
Hypertension (HT)	233 (31.5)	184 (31.1)	49 (33.1)	0.635
Diabetes mellitus (DM)	113 (15.5)	69 (12.1)	44 (28.2)	<0.001
Hyperlipidemia (HL)	56 (7.6)	36 (6.2)	20 (12.8)	0.009
Peripheral artery disease	7 (1)	6 (1)	1 (0.7)	1.000
Preop chronic obstructive pulmonary disease (COPD)	63 (9.4)	47 (8.8)	16 (11.7)	0.383
Preop GI bleeding	9 (1.2)	7 (1.2)	2 (1.3)	1.000
Preop cerebrovascular event (CVA)	48 (6.5)	39 (6.7)	9 (5.9)	0.853
Preop hemodialysis	11 (1.6)	10 (1.9)	1 (0.7)	0.473
Pro-brain natriuretic peptide (Pro-BNP)	4909.5 (0–70,000)	4758 (0–70,000)	5400 (0–39,716)	0.895
0–1000	33 (10)	26 (10.1)	7 (9.6)	0.875
1000–5000	134 (40.6)	106 (41.2)	28 (38.4)	
>5000	163 (49.4)	125 (48.6)	38 (52.1)	
Creatinine	1 (0–109)	1.1 (0–109)	1 (0–17.2)	0.111
Bilirubin	1.2 (0–28)	1.2 (0–28)	1.4 (0–7.9)	0.843
International Normalized Ratio (INR)	1.2 (0–15)	1.2 (0–15)	1.2 (0–3.2)	0.871
Albumin	3.9 (0–46.5)	4 (0–46.5)	3.9 (0–43.1)	0.916
Hemoglobine	12 (0–137)	12 (0–137)	11.8 (0–16.9)	0.328
Preop leukocyte	8.1 (0–84.7)	8.2 (0–84.7)	7.9 (0–20.1)	0.160

**Table 2:** Comparison of Preoperative Characteristics in Patients With and Without Driveline Infection.

Variable	Total (*n* = 772)	No DLI (*n* = 614)	DLI (*n* = 158)	*p* Value
Preoperative lactate (median, range)	1.4 (0–73)	1.4 (0–73)	1.4 (0–6)	0.970
INTERMACS profile, *n* (%)				0.851
1	65 (8.7)	54 (9.1)	11 (7.1)	
2	238 (31.9)	190 (32.1)	48 (31.0)	
3	278 (37.3)	218 (36.9)	60 (38.7)	
4	159 (21.3)	125 (21.2)	34 (21.9)	
5	6 (0.8)	4 (0.7)	2 (1.3)	
Redo cardiac surgery, *n* (%)	130 (17.2)	109 (18.1)	21 (13.4)	0.159
Preoperative inotrope use, *n* (%)	455 (62.2)	359 (62.3)	96 (61.9)	0.929
Preoperative IABP, *n* (%)	52 (7.1)	41 (7.0)	11 (7.1)	1.000
Preoperative ECMO, *n* (%)	6 (0.8)	5 (0.9)	1 (0.6)	1.000

**Table 3:** Comparison of ventricular assist devices in terms of driveline infection.

	Total	No Driveline Infection (*n* = 614)	with Driveline Infection (*n* = 158)	*p*-Value
	*n* (%)	*n* (%)	*n* (%)	
Types of devices				0.001
HeartWare (HVAD)	489 (100%)	405 (82%)	84 (18%)	
HeartMate 2	171 (100%)	134 (78%)	37 (22%)	
HeartMate 3	106 (100%)	71 (66%)	35 (34%)	

**Table 4:** Comparison of surgical method, cardiopulmonary bypass (CPB) time, cross-clamp (X-Clamp) time, and additional surgery during the operation.

	Total	No Driveline Infection (*n* = 614)	with Driveline Infection (*n* = 158)	*p*-Value
	*n* (%) or Median (Min–Max)	*n* (%) or Median (Min–Max)	*n* (%) or Median (Min–Max)	
Surgical method				
Thoracotomy	90 (11.8)	72 (11.9)	18 (11.5)	1.000
Sternotomy	677 (88.4)	537 (88.2)	140 (89.2)	0.836
Minimally Invasive	12 (1.6)	11 (1.8)	1 (0.6)	0.476
CPB	758 (99.3)	602 (99.3)	156 (99.4)	1.000
CPB time	63 (32–298)	63 (32–298)	63 (37–196)	0.761
X-clamp	36 (4.8)	28 (4.7)	8 (5.2)	0.980
X-clamp time	60 (3–115)	60 (3–115)	61 (19–110)	0.947
Additional surgery during the operation				
Tricuspid valve repair	39 (5.4)	28 (4.9)	11 (7.3)	0.331
Aortic valve replacement	11 (1.5)	8 (1.4)	3 (1.9)	0.707
Aortic replacement	21 (2.8)	16 (2.7)	5 (3.2)	0.785

**Table 5:** Comparison of postoperative revision, long-term complications, short-time complications, right heart failure, RVAD long-term, temporary RVAD (Levitronix or ECMO), postop nitric oxide, total support time (day), heart transplant, and exitus.

	Total	No Driveline Infection (*n* = 614)	with Driveline Infection (*n* = 158)	*p*-Value
	*n* (%) or Median (Min–Max)	*n* (%) or Median (Min–Max)	*n* (%) or Median (Min–Max)	
Hemorrhage Revision	60 (8.1)	54 (9.2)	6 (3.8)	0.045
Postoperative right heart failure	42 (7.1)	39 (8.2)	3 (2.6)	0.057
Postop Nitric oxide	131 (18.5)	115 (20.5)	16 (10.8)	0.010
Postoperative inotropic support for more than 14 days	23 (3.3)	21 (3.8)	2 (1.4)	0.194
Temporary RVAD (Levitronix or ECMO)	20 (2.8)	20 (3.5)	0 (0)	0.012
RVAD long term	13 (1.8)	12 (2.1)	1 (0.7)	0.322
Postoperative complication				
Acute kidney failure	9 (1.4)	9 (1.7)	0 (0)	0.216
Hemodialysis	27 (4.1)	27 (5.1)	0 (0)	0.013
Sepsis	61 (8.7)	51 (9.2)	10 (6.9)	0.477
Intensive care stay (Day)	5 (1–60)	5 (1–60)	5 (2–60)	0.038
Long-term complications				
GI bleeding	73 (11.6)	55 (11.3)	18 (12.9)	0.720
Ischemic stroke	116 (18.3)	98 (19.8)	18 (12.9)	0.066
Hemorrhagic Stroke	104 (16.4)	85 (17.3)	19 (13.5)	0.345
Pump thrombosis	177 (27.4)	130 (25.9)	47 (32.6)	0.110
Total support time (day)	978(1–4752)	859 (1–4752)	1995.5 (7–4684)	<0.001
Heart transplant	83 (14)	74 (15.9)	9 (7.1)	0.018

**Table 6:** Multivariable analysis of independent predictors of driveline infection.

Variables	OR (%95 CI)	*p*-Value
Age	0.996 (0.956–1.037)	0.837
Gender		
Male	Reference	-
Female	0.756 (0.240–2.385)	0.634
Body mass index (BMI)	1.095 (0.994–1.207)	0.066
Diabetes mellitus (DM)	3.298 (1.292–8.417)	0.013
Types of devices		
HeartWare (HVAD)	Reference	-
HeartMate 2	0.740 (0.252–2.171)	0.583
HeartMate 3	1.496 (0.552–4.052)	0.428
Hemorrhage Revision	0.294 (0.020–4.350)	0.373
Postop Nitric oxide	0.517 (0.151–1.773)	0.294
Intensive care stay (Day)	1.010 (0.953–1.071)	0.728
Total support time (day)	1.000 (1.000–1.001)	0.003

**Table 7:** Demographic characteristics of patients with drive-line infection and distribution of submitted culture samples.

Variables (*n* = 154)	*n* (%)	Mean ± SD	Median (Min–Max)
Age (years)		51 ± 13.5	53 (7–71)
Gender			
Male	126 (79.7)		
Female	32 (20.3)		
Collected Sample		11 ± 8.6	8 (1–30)
Sample type			
Abscess	19 (1.2)		
Sputum	38 (2.3)		
Urine	158 (9.7)		
Blood culture	293 (18.0)		
Tissue/Biopsy	10 (0.6)		
Deep tracheal aspiration	71 (4.4)		
Tissue swab culture	1035 (63.7)		

**Table 8:** The Most Common Pathogens in Patients and the Distribution of Antibiotic Resistance.

Pathogens	Number of Samples with Bacterial Growth	Ciprofloxacin	MRSA	Piperacillin/ Tazobactam	Penicillin	Imipenem
		Antibiotic Resistance Sample = 381	Antibiotic Resistance Sample = 174	Antibiotic Resistance Sample = 172	Antibiotic Resistance Sample = 154	Antibiotic Resistance Sample = 134
*Alcaligenes faecalis* ssp. faecalis	4	2	0	0	3	0
*Achromobacter xylosoxidans*	7	1	0	0	5	0
*Acinetobacter johnsonii*	1	0	0	0	0	0
*Acinetobacter baumannii*	35	30	0	7	1	28
*Acinetobacter haemolyticus*	2	0	0	0	0	0
*Achromobacter denitrificans*	1	0	0	0	0	0
*Burkholderia cepacia*	2	0	0	0	0	0
*Burkholderia contaminans*	2	0	0	0	0	0
*Citrobacter freundii*	1	0	0	0	0	0
*Candida albicans*	14	0	0	0	0	0
*Corynebacterium accolens*	1	0	0	0	0	0
*Corynebacterium amycolatum*	34	27	0	1	27	1
*Corynebacterium aurimucosum*	3	3	0	0	3	0
*Corynebacterium striatum*	64	51	0	1	54	1
*Corynebacterium simulans*	4	2	0	0	2	0
*Corynebacterium tuberculostearicum*	3	3	0	0	2	0
*Corynebacterium jeikeium*	3	3	0	0	3	0
*Corynebacterium xerosis*	2	2	0	0	2	0
*Citrobacter koseri*	5	0	0	0	0	0
*Dermabacter hominis*	1	1	0	0	1	0
Broad-spectrum beta-lactamase	18	15	0	12	1	0
*Enterococcus raffinosus*	1	1	0	1	0	0
*Enterococcus avium*	1	0	0	0	0	0
*Enterobacter cloacae* ssp. cloacae	12	0	0	0	0	0
*Enterococcus faecalis*	44	0	0	0	0	0
*Enterococcus faecium*	18	6	0	0	0	0
*Escherichia coli*	103	9	0	6	0	0
*Haemophilus parainfluenzae*	1	0	0	0	0	0
*Haemophilus influenzae* non tip b	8	0	0	0	0	0
*Coagulase-Negative Staphylococcus* (*CNS*)	58	2	48	0	0	0
*Klebsiella pneumoniae*	98	7	0	6	0	5
*Moraxella* (*Branhamella*) *catarrhalis*	2	0	0	0	0	0
*Morganella morganii*	5	0	0	0	0	0
*Providencia stuartii*	1	0	0	0	0	0
*Proteus mirabilis*	28	0	0	0	0	1
*Pseudomonas putida*	1	1	0	1	0	1
*Pseudomonas aeruginosa*	323	193	0	131	1	97
*Stenotrophomonas maltophilia*	16	0	0	0	0	0
*Streptococcus mitis*	5	0	0	0	2	0
*Streptococcus oralis*	5	0	0	0	0	0
*Staphylococcus haemolyticus*	9	0	7	0	0	0
*Staphylococcus hominis*	10	0	7	0	0	0
*Staphylococcus aureus*	590	6	90	1	44	0
*Streptococcus vestibularis*	1	0	0	0	1	0
*Streptococcus agalactiae*	4	0	0	0	0	0
*Streptococcus sanguinis*	1	0	0	0	1	0
*Streptococcus pneumoniae*	6	0	0	0	0	0
*Staphylococcus epidermidis*	40	1	22	0	1	0
*Serratia marcescens*	61	15	0	5	0	0

## Data Availability

The data are available within the article.

## Abbreviations

VAD	Ventricular assist device
BTT	bridge to transplant
DT	destination therapy
CVA	cerebrovascular accident
DLI	driveline infection
LVAD	left ventricular assist device
BMI	body mass index
INR	international normalized ratio
INTERMACS	Interagency Registry for Mechanically Assisted Circulatory Support
NT-proBNP	N-terminal pro-brain natriuretic peptide
IABP	intra-aortic balloon pump
ECMO	extracorporeal membrane oxygenation
CPB	cardiopulmonary bypass
ICU	intensive care unit
RVAD	right ventricular assist device
MDR	multidrug-resistant
MSSA	methicillin-susceptible *Staphylococcus aureus*
MRSA	methicillin-resistant *Staphylococcus aureus*
MR-CoNS	methicillin-resistant coagulase-negative staphylococci MR-CoNS
